# A fully automatic algorithm for assessing T2* and its certainty value for accurate cardiac and liver iron load determination

**DOI:** 10.1186/1532-429X-15-S1-P141

**Published:** 2013-01-30

**Authors:** Erik Hedstrom, Einar Heiberg, Gerald F Greil, Eike Nagel

**Affiliations:** 1 Cardiac MR Group, Dept of Radiology and Clinical physiology, Lund University and Skane University Hospital, Lund, Sweden; 2 Division of Imaging Sciences and Biomedical Engineering, King's College London, London, UK; 3 BHF Centre of Research Excellence and NIHR Biomedical Research Centre at Guy's and St Thomas' NHS Foundation Trusts and King's College London, London, UK

## Background

Quantification of myocardial and liver iron load has become a mainstay of guiding therapy in thalassaemia patients. However, current quantification methods are user dependent for data-point exclusion before curve fitting, and do not report the T2* certainty. Recently, an automatic inline maximum likelihood estimate (MLE) method with k-space Rician noise correction was validated against the reference standard manual truncation method. We now present a vendor-independent offline tool for fully automatic post processing and T2* quantification with certainty estimates, tested in computer simulations and patients with iron overload.

## Methods

A new hybrid method was implemented in Segment (http://segment.heiberg.se), combining exponential fitting and residual-weighting. The only user dependency is drawing the ROI for evaluation.

Hybrid method: One of three fitting algorithms is applied based on an initial automated T2* estimation. If this estimate is shorter than the first echo time (TE_1_), an exponential model is used with offset correction and certainty residual-weighting; if the estimate is longer than 3×TE_1_, a pure exponential model residual-weighted to certainty of pixels is used; and if the estimate falls in-between TE1 and 3×TE_1_, a linear interpolation of the two methods is applied. Possible bias for large T2* values (>>maximum TE) is compensated for by using the magnitude of the fit residual error. Certainty estimates of T2* are calculated based on size of the fit residual error. For low T2* value certainty estimates the TEs are also taken into account.

Simulations: Computer phantoms were generated for T2*=0.5-40 ms with varying Rician noise. For each T2* and noise level 200 computer phantoms were created for calculation of T2* certainty.

Patient study: Nine iron overload patients (5 male; median age 12, range 1.4-39 years) were scanned at 1.5T for cardiac and liver iron load using 2 multi-gradient echo sequences. All parameters were identical (voxel = 2 × 2 × 10 mm, matrix = 256, 10 echoes, FA = 20°, BW = 833 Hz, SENSE = 2) except for TE (cardiac: TE_1_/ΔTE 2.5/2.3 ms; liver: TE_1_/ΔTE 0.8/1.6 ms). The new hybrid method and the MLE method were applied in the cardiac septum and liver, respectively.

## Results

**Figure 1:**
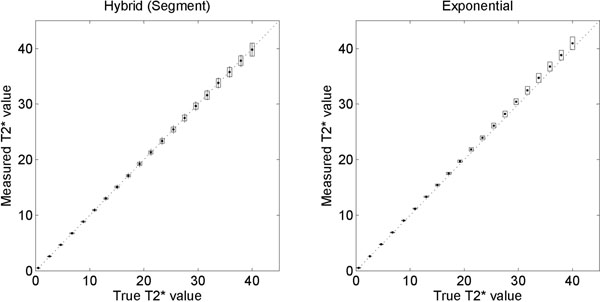
Hybrid method (left) and standard fitting (right) both agree well with the theoretical T2* values from computer simulation phantoms. Boxes indicate measured uncertainty and vertical lines indicate calculated uncertainty (Hybrid method only). Note the trend of overestimating the true T2* value with increasing T2* for the commonly used exponential fitting method (right).

**Figure 2:**
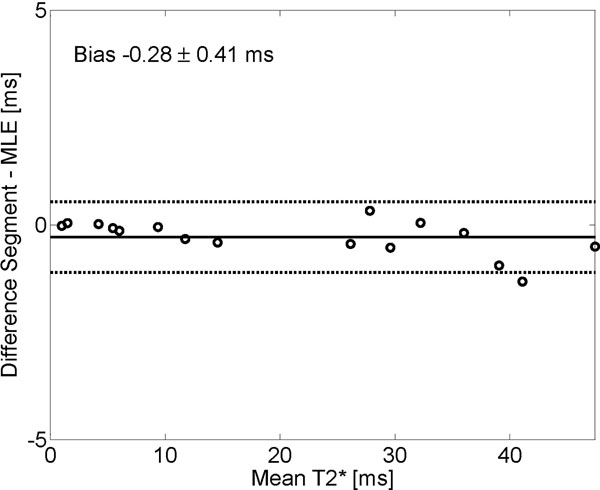
In patients, segment and MLE are comparable over a wide range of T2* values.

Results of computer phantom experiments are shown in Figure 1. In patients, bias ± SD between Segment and MLE were -0.28±0.41 ms (-1.16 ± 1.68%; Figure 2).

## Conclusions

Accurate iron-load T2* is provided by the proposed fully automatic method, comparable with the MLE method over a wide range. In addition to previous algorithms, the new hybrid algorithm reports a certainty estimate for T2*.

## Funding

Swedish Research Council grants VR 621-2008-2949 and VR K2009-65X-14599-07-3; National Visualization Program and Knowledge Foundation grant 2009-0080; the Medical Faculty at Lund University, Sweden; the Region of Scania, Sweden; Skane University Hospital, Lund, Sweden; The Foundation BLANCEFLOR Boncompagni-Ludovisi, née Bildt; the Swedish Societies of Medicine, Radiology, and Cardiology; Covidien, Sweden; and the Swedish Heart Lung Foundation; the British Heart Foundation, UK; and the Biomedical Research Centre, UK.

